# Nanoscale Characterization of Interaction of Nucleosomes with H1 Linker Histone

**DOI:** 10.3390/ijms26010303

**Published:** 2024-12-31

**Authors:** Ahmed Yesvi Rafa, Shaun Filliaux, Yuri L. Lyubchenko

**Affiliations:** Department of Pharmaceutical Sciences, University of Nebraska Medical Center, Omaha, NE 68198-6025, USA; arafa@unmc.edu (A.Y.R.); shaun.filliaux@unmc.edu (S.F.)

**Keywords:** Nucleosome, chromatosome, H1 linker histone, chromatin compaction, atomic force microscopy (AFM), gel electrophoresis

## Abstract

In eukaryotic nuclei, DNA is wrapped around an octamer of core histones to form nucleosomes. H1 binds to the linker DNA of nucleosome to form the chromatosome, the next structural unit of chromatin. Structural features on individual chromatosomes contribute to chromatin structure, but not fully characterized. In addition to canonical nucleosomes composed of two copies each of histones H2A, H2B, H3, and H4 (H3 nucleosomes), centromeres chromatin contain nucleosomes in which H3 is replaced with its analog CENP-A, changing structural properties of CENP-A nucleosomes. Nothing is known about the interaction of H1 with CENP-A nucleosomes. Here we filled this gap and characterized the interaction of H1 histone with both types of nucleosomes. H1 does bind both types of the nucleosomes forming more compact chromosome particles with elevated affinity to H3 nucleosomes. H1 binding significantly increases the stability of chromatosomes preventing their spontaneous dissociation. In addition to binding to the entry-exit position of the DNA arms identified earlier, H1 is capable of bridging of distant DNA segments. H1 binding leads to the assembly of mononucleosomes in aggregates, stabilized by internucleosome interactions as well as bridging of the DNA arms of chromatosomes. Contribution of these finding to the chromatin structure and functions are discussed.

## 1. Introduction

Nucleosomes are the basic structural unit of chromatin, consisting of approximately 147 base pairs of DNA wrapped around a histone octamer, composed of two copies each of histone H2A, H2B, H3, and H4 [1,2,3,4,5,6]. This packaging is fundamental to genome organization, condensing the DNA and regulating essential cellular processes such as transcription, DNA replication, and repair [7,8,9,10,11]. The structural configuration of the nucleosome is highly dynamic contributing to the DNA accessibility to various regulatory proteins of gene expression [12,13,14,15,16,17].

Histone H1, also known as the linker histone, binds to the nucleosome forming the chromatosome, stabilizes the nucleosome and facilitates assembly of higher-order chromatin structures [18,19,20,21,22,23]. In the chromatosome, extra ~20 extra base pairs of linker DNA are included in the chromatosome core structure increasing the size of wrapped DNA [24,25,26]. H1’s globular domain connects with the nucleosome at the dyad axis, and its positively charged C-terminal tail binds to linker DNA, stabilizing its conformation [27,28,29,30]. The length and flexibility of linker DNA have a substantial impact on H1 binding dynamics, with longer linker DNA allowing for more asymmetric and varied interactions that affect chromatin compaction [31,32,33,34].

In addition to canonical nucleosomes consisting of the octamer H2A, H2B, H3, and H4 (H3 nucleosomes), the centromeric chromatin, which are specialized segments of chromosomes that aid chromosomal segregation after DNA replication contain a modified H3 histone called the centromere protein A (CENP-A) [35,36,37,38,39]. Crystallographic studies reveal that replacing canonical H3 with CENP-A retains the octameric nucleosome structure and the overall DNA wrapping around the histone core but reduces the length of the wrapped DNA compared to H3 nucleosomes [40]. Furthermore, specific structural and biochemical properties unique to CENP-A nucleosomes have been characterized, including reduced DNA wrapping length, altered histone-DNA interactions, and distinct centromeric protein binding affinities, highlighting their specialized role in centromere function [41,42,43,44,45,46,47], but no data on the effect of H1 linker of CENP-A nucleosome properties are available. Structural characterization of CENP-A nucleosomes and chromatosomes is essential for the formation of the kinetochore, a protein complex required for chromosome segregation during mitosis. Therefore, understanding how linker histone H1 influences centromeric chromatin, and how its effects differ between H3 and CENP-A nucleosomes, is critical for elucidating the broader role of chromatin compaction in maintaining genome stability.

In this study, we characterized the interactions of H3 and CENP-A nucleosomes with H1 linker histone using the DNA template with arms as long as ~100 bp. We utilized Atomic Force Microscopy (AFM) and gel electrophoresis to probe the structural effects of H1 binding on both canonical H3 nucleosomes and CENP-A nucleosomes. Gel electrophoresis revealed the effect of H1 on the migration of both types of nucleosomes and the AFM studies showed that this effect can be explained by compaction of nucleosomes. AFM studies also revealed the ability of H1 to bridge DNA segments away from the nucleosome core, the feature that contributes to the chromatosomes compaction. Bridging binding mode of H1 can explain the formation of larger aggregates assembled only in the presence of H1 histone visualized with AFM directly.

## 2. Results

**Figure 1:**
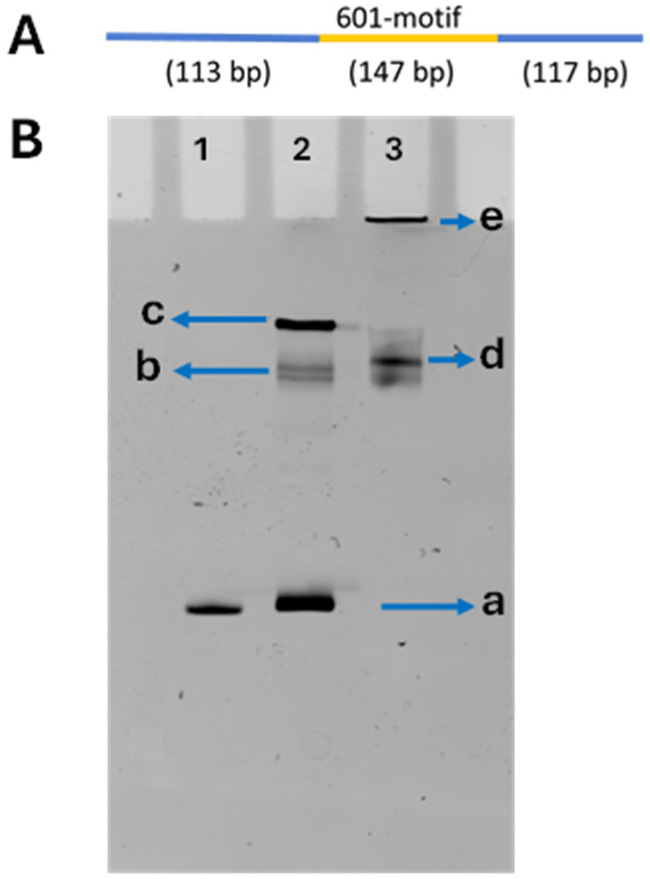
DNA schematic and the gel electrophoresis data (**A**) DNA schematic**.** The full length of the 377 bp DNA substrate. The central 147 bp Widom 601 sequence is shown in yellow, flanked by 113 bp and 117 bp DNA arms from the plasmid in which 601 motif was cloned; they have with no specificity to nucleosomes assembly. (**B**) Native-PAGE analysis of H3 nucleosomes and chromatosomes with EtBr staining. Lane 1: DNA; Lane 2: H3 nucleosomes; Lane 3: H3 chromatosomes. Band (a) points to the position of free DNA. Bands (b) and (c) correspond to mobilities of nucleosome. Band (d) is chromatosome and band (e) points to aggregates formed in the in chromatosome sample.

In this study, we designed a 377 bp DNA substrate in which the central nucleosome-specific 147 bp Widom 601 motif is flanked by 113 bp and 117 bp DNA segments on the left and right, respectively (Figure 1A). Two types of nucleosomes were assembled on this substrate: canonical nucleosomes containing the octamer assembled with H2A, H2B, H3, and H4 histones and H2A, H2B, H4 histones and the centromere (CENP-A histone, termed as nucleosomes H3 and CENP-A, respectively (molar ratio of DNA and histone octamers was 1:1). Assembled nucleosomes were mixed with the linker histone H1 in 1:1:2 for the DNA:(histone octamer):H1 molar ratios to produce H3 and CENP-A chromatosomes as described in the methods section.

### 2.1. Characterization of H3 Nucleosomes and Chromatosomes

#### 2.1.1. Gel Electrophoresis

The assembly of nucleosomes and chromatosomes was monitored by native-PAGE gel; a typical gel is shown in Figure 1B. The nucleosome sample (lane 2), in addition to the fast-migrating band (a) with the position coinciding with that of free DNA (lane 1), is characterized by two additional bands (b and c), which are assigned to the bands of assembled nucleosomes. Analysis of scans of the gel (Figure S1) showed that band (b) represents 22%, and band (c) accounts for 28% of the total volume. The free DNA band (a) is 50% of the total volume. The gel electrophoresis pattern of chromatosomes (lane 3) differs from the one for nucleosomes. The chromatosomes, instead of two bands for nucleosomes (lane 2), migrate as one major diffused band (d) moves faster than band (c) for the nucleosome sample. There were no free DNA bands like the band (a) in lanes 1 and 2 (Figure S2). These findings suggest that H1 histones stabilize nucleosomes, preventing their dissociation during dilution for the preparation of samples [48]. A band (e) at the top of the gel pointed to the formation of aggregates not entering the gel. Their yield according to (Figure S2) is 28%.

#### 2.1.2. AFM Characterization of H3 Nucleosomes and Chromatosomes

**Figure 2:**
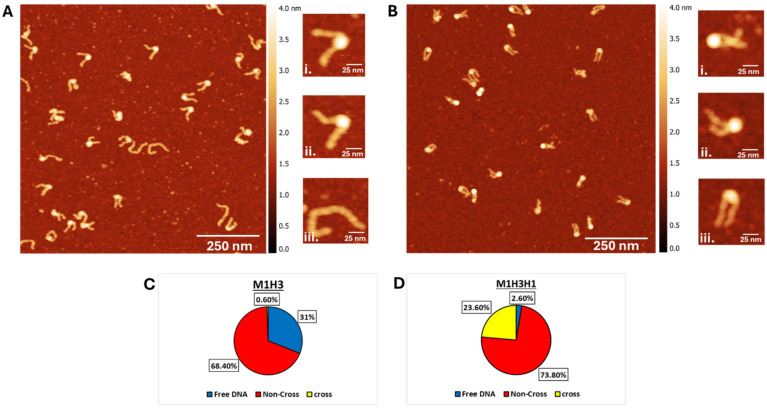
AFM images and Subpopulation Distribution: (**A**,**B**) AFM images of H3 nucleosomes (**A**) and H3 chromatosomes (**B**). Zoomed images (**i**) and (**ii**) in (**A**) show examples of H3 nucleosomes with separated DNA arms, while (**iii**) represents free DNA. In (**B**), H3 chromatosomes are shown, with zoomed images (**i**) and (**ii**) highlighting crossed DNA arms, and also (**iii**) depicting a compact molecule configuration, which was not observed in H3 nucleosomes. The above images are 1000 × 1000 nm scans at 1024 pixels per line. (**C**,**D**) Subpopulation distribution of H3 nucleosome and H3 chromatosome. Blue represents free DNA, and red & yellow represent non-cross & cross, respectively. Chromatosomes show a significant increase in crossed DNA arms and a dramatic reduction in free DNA, reflecting enhanced stability and compaction due to H1.

The samples were deposited on functionalized mica substrates, dried, and imaged with AFM. Typical 1000 nm × 1000 nm images for H3 nucleosomes and chromatosomes are shown in Figure 2A and Figure 2B, respectively. The H3 nucleosome sample appears with bright globular features flanked with separated DNA arms, which corresponds to the nucleosome assembly on such substrate [49]. Zoomed-selected images of individual nucleosomes are shown to the right of Figure 2A. Images (i) and (ii) are relatively similar assemblies with arms separated from each other. Image (iii) corresponds to naked DNA, and such assemblies are easily identified in Figure 2A.

Images of chromatosomes (Figure 2B) are different. Histone cores appear as bright globular features in these images, but the arms are not separated and can be crossed. A zoomed image of one of such crossed complexes (i) is to the right of the main image in Figure 2B. Image (ii) illustrates species in which DNA 2 arms are not crossed but moved not far and image (iii) belongs to species in which the arms are separated but very close. Several parameters were measured to characterize differences in the nanoscale structure of nucleosomes and chromatosomes.

##### Morphologies of H3 Nucleosomes and Chromatosomes

Zoomed image (i) & (ii) are the H3 nucleosomes in Figure 2A. Additionally, free DNA appears on the AFM images (Figure 2A-zoomed image (iii). The yield of free DNA calculated over the large set of images is 31% (Figure 2C), which is consistent with the yield of free DNA in gel results (50%, Figure 1B, lane 2, band “a”).

A similar analysis was done for chromatosome samples; the data are shown in Figure 2D. One distinct feature of chromatosome is the formation of assemblies with crossed arms, as shown in the image (i) in Figure 2B. The population of such species is 23.6%, with non-crossed DNA arms as the primary species of chromatosomes (73.8%). No crossed assemblies were observed in the control DNA substrate, which was complexed with H1. Only a small fraction (0.6%) of the H3 nucleosome sample showed crossed DNA arms, suggesting minimal crossing the individual DNA flank arms in the absence of H1. Free DNA molecules are a very rare species for the H3 chromatosome. No such molecules are seen in Figure 2B, but they appear in other images with the overall yield as low as 2.6%. This finding is consistent with the gel results in which no free DNA was detected for the chromatosome sample (Figure 1, lane 3).

##### Comparative Characterization of H3 Nucleosomes and Chromatosomes

Several parameters characterizing the nanoscale structure of nucleosomes and chromosomes were measured to reveal the effect of H1 histone on the chromatosome structure at nanoscale.

**Figure 3:**
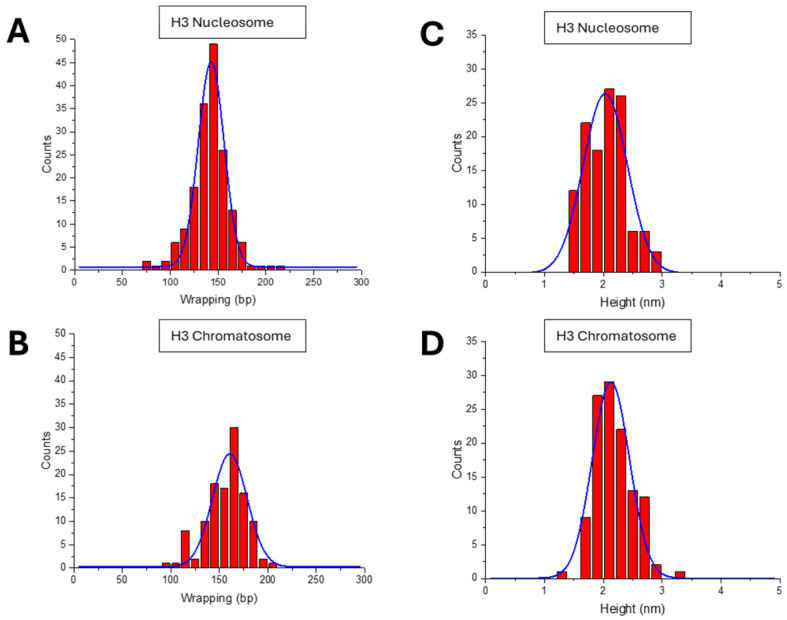
Results for the DNA wrapping lengths and the nucleosome particle sizes measurements: (**A**,**B**) Histograms show DNA wrapping efficiency for H3 nucleosomes (143 ± 16 bp) and H3 chromatosomes (161 ± 21 bp), highlighting the increased wrapping with H1. (**C**,**D**) Histograms of nucleosomal core particle height for H3 nucleosomes (2.0 ± 0.4 nm) and H3 chromatosomes (2.1 ± 0.4 nm), showing minimal changes in core height upon addition of H1.

The length of DNA wrapped around the nucleosome particle was one of such features. It was obtained by subtracting the arms’ lengths from the free DNA’s contour length. The data on the length of the DNA wrapped around the histone core for the nucleosome assembled as a histogram are shown in Figure 3A. The distribution is narrowed around 143 ± 16 bp, consistent with the expected 147 bp value for 601 DNA substrate [50]. A similar analysis was performed for H3 chromatosomes. The data in Figure 3B demonstrates a dramatic effect of H1 binding. The wrapping efficiency increased significantly, with 161 ± 21 bp of DNA, which is higher than the size of wrapped DNA for nucleosomes and in line with published results [18,24,51]. The *p*-value comparing the wrapping efficiency between H3 nucleosomes and chromatosomes was 8.78 × 10^−9^, indicating a statistically significant difference.

The height of the nucleosomal core particle was measured, and the data are shown in Figure 3C. Nucleosomes exhibited an average core particle height of 2.0 ± 0.4 nm. A similar analysis of the H3 chromatosome was done, and the data shown in Figure 3D produces the histogram approximated with a Gaussian with a maximum of 2.1± 0.4 nm. The results indicated to a small difference in core particle height between nucleosomes and chromatosomes. The *p*-value for the difference in the height of the nucleosomal core particle between H3 nucleosomes and chromatosomes was 0.074, suggesting the difference, although not very significant.

A visual inspection of AFM images in Figure 2A,B reveals a dramatic difference in the shape of nucleosomes and chromatosomes. The latter appear on the AFM images as more compact complexes. Zoomed selected images to the right of Figure 2A,B illustrate it. A set of parameters was measured to characterize this visual difference structurally.

**Figure 4:**
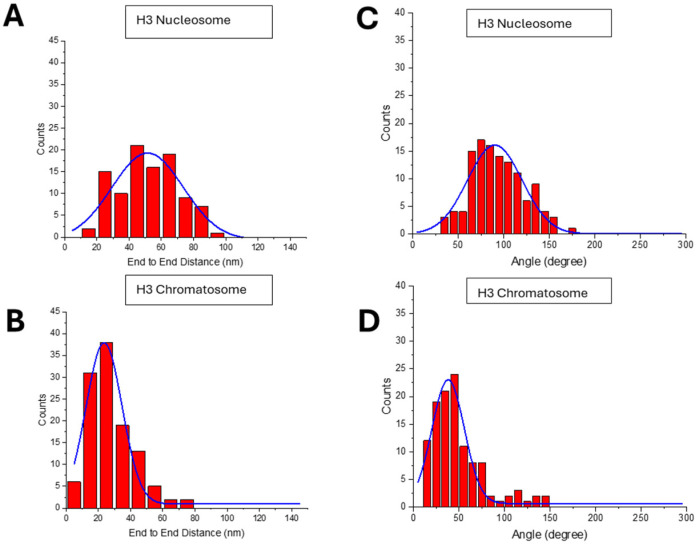
Results for the end-to-end distance and angle measurements: (**A**,**B**) End-to-end distance measurements for H3 nucleosomes (52 ± 22 nm) and H3 chromatosomes (24 ± 13 nm). (**C**,**D**) The angle between DNA arms for H3 nucleosomes ((**C**): 90 ± 35°) and chromatosomes ((**D**): 38 ± 18°). Chromatosomes have a lower DNA entry/exit angle indicating that H1 reduces the angles and nucleosome compaction.

The end-to-end distance is one of such parameters. The histogram of this parameter shown in Figure 4A demonstrates that this parameter changes in a broad range with a maximum in the Gaussian with 52 ± 22 nm. The large range indicates a high dynamic of opening of the nucleosome flanks. Results of similar analysis for H3 chromatosomes are shown in Figure 4B. The distribution is shifted to smaller values, producing the distance between the flanks of chromatosomes as low as 24 ± 13 nm.

Another parameter characterizing the effect of H1 histone is the angle between the DNA arms, which was measured at the close vicinity to the nucleosome core. The data in Figure 4C show that the Gaussian distribution is 90 ± 35°. Similarly, the angle between the flanks for H3 chromatosomes was measured. The data are assembled in Figure 4D. The histogram is narrow with the maximum value of the angle 38 ± 18°, which is considerably less than the angle values for H3 nucleosomes, 90 ± 35°. The *p*-value for the angle between the two DNA arms in H3 nucleosomes and chromatosomes was 3.02 × 10^−18^. This significant reduction suggests a dramatic compaction of the DNA arms of the nucleosome induced by H1 linker histone.

### 2.2. Characterization of CENP-A Nucleosomes and Chromatosomes

#### 2.2.1. Gel Electrophoresis

**Figure 5:**
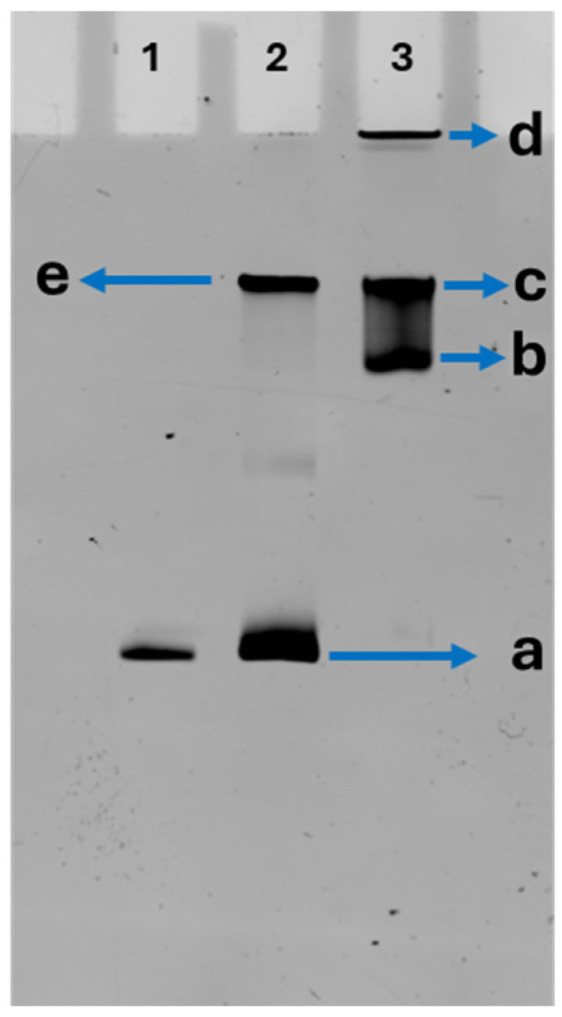
Native gel electrophoresis results: 5% polyacrylamide native-PAGE for CENP-A nucleosomes and chromatosomes with EtBr staining; Lane 1: DNA, Lane 2: CENP-A Nucleosome, Lane 3: CENP-A Chromatosomes. Arrows to bands (a, b c, d and e) point to positions of free DNA, nucleosomes, chromatosomes, and aggregates of chromatosomes, respectively. Chromatosomes exhibit distinct gel mobility patterns due to H1 binding.

Similar to studies of H3 nucleosomes, the assembly of CENP-A nucleosomes and chromatosomes was monitored by native gel electrophoresis, and a typical gel image is shown in Figure 5. The CENP-A nucleosome sample (lane 2) displayed a fast-migrating band (a) that aligned with the free DNA band (lane 1) yield of which according to the scans of the gel (Figure S3) is 63% with the yield of nucleosome (band e) 37%. The gel electrophoresis pattern for CENP-A chromatosomes (lane 3) differ from nucleosomes, showing a broad, diffuse bands between relatively sharp bands (b,c) as supported by scanning data of the gel in the supplemental Figure S4. The position of band (c) coincides with the one for the nucleosome (band (e) in lane 2) but band (b) corresponds to the faster migrating fraction of chromatosome CENP-A sample. The yield of band (c) and (b) are 42% and 30%, respectively (Figure S4). No free DNA is detected in the chromatosome CENP-A sample as also observed for H3 chromatosome samples (Figure 1B). An additional band (d) near the top of the gel points to the formation of aggregates that were unable to enter the gel, which is 12% of total yield (Figure S4)

#### 2.2.2. AFM Characterization of CENP-A Nucleosomes and Chromatosomes

**Figure 6:**
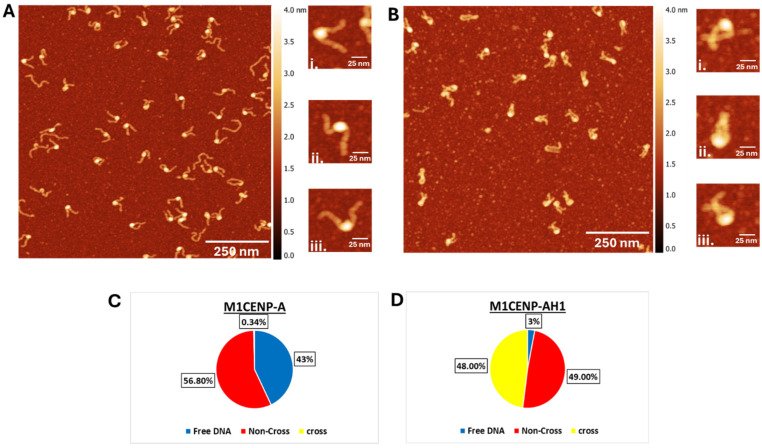
AFM images and subpopulation distribution: (**A**,**B**) AFM images (1000 × 1000 nm) of CENP-A nucleosomes (**A**) and CENP-A chromatosomes (**B**). Chromatosomes exhibit increased compaction and a higher frequency of crossed DNA arm configurations compared to nucleosomes. Zoomed images (**i**), (**ii**), and (**iii**) in (**A**) show examples of CENP-A nucleosomes with separated DNA arms. In (**B**), zoomed images (**i**), (**ii**), and (**iii**) highlight crossed DNA arms, depicts a compact molecule configuration not observed in CENP-A nucleosomes. The above images are 1000 × 1000 nm scans at 1024 pixels per line. (**C**,**D**) Subpopulation distribution of CENP-A nucleosome and CENP-A chromatosome. Blue represents free DNA, and red & yellow represents non-cross & cross respectively. H1 enhances chromatosome stability and compaction, as reflected by reduced free DNA and increased crossed DNA arm configurations.

Typical 1000 nm × 1000 nm images of CENP-A nucleosomes and chromatosomes are shown in Figure 6A and Figure 6B, respectively. The CENP-A nucleosome sample displayed bright, globular features with separated DNA arms, indicating successful nucleosome assembly on the substrate. Zoomed images of individual nucleosomes with clearly separated arms are shown to the right of Figure 6A.

Images of CENP-A chromatosomes are shown in Figure 6B. The nucleosome cores appearing as bright globular features but the DNA arms often closer together or crossed. A close-up of a chromatosome with crossed arms is shown in image (i) to the right of Figure 6B. Additional examples of arm configurations are shown in images (ii) and (iii), reflecting variations in DNA arm spacing. Parameters were measured to quantitatively characterize the nanoscale structural differences between CENP-A nucleosomes and chromatosomes.

##### Morphologies of CENP-A Nucleosomes and Chromatosomes

AFM images of CENP-A nucleosomes showed both free DNA and nucleosome-bound complexes. Free DNA molecules were marked in the images and comprised 57% of observed particles in the CENP-A nucleosome sample (Figure 6A), consistent with the gel results (Figure 5, lane 2). This number is consistent with the gel image according to which the yield of free DNA is 63% (Figure S3). In contrast, free DNA (Figure 6B) are rare species for the CENP-A chromatosome sample, making up only 3% of observed particles. Such low yield of free DNA is consistent with the gel electrophoresis data, which did not detect free DNA in the chromatosome sample (Figure 5, lane 3). This reduction suggests that the H1 linker histone enhances nucleosome stability by minimizing the spontaneous dissociation of DNA. As illustrated in AFM images (Figure 6B), the chromatosomes DNA arms frequently crossed due to H1 incorporation. The analysis of multiple images revealed that 48% of chromatosomes displayed crossed DNA arms, compared to only 0.34% in CENP-A nucleosomes.

##### Comparative Characterization of CENP-A Nucleosomes and Chromatosomes

Similar to previous analyses for H3 samples, structural differences between CENP-A nucleosomes and chromatosomes were characterized by measuring DNA wrapping length, core particle height, end-to-end distance, and the angle between DNA arms.

**Figure 7:**
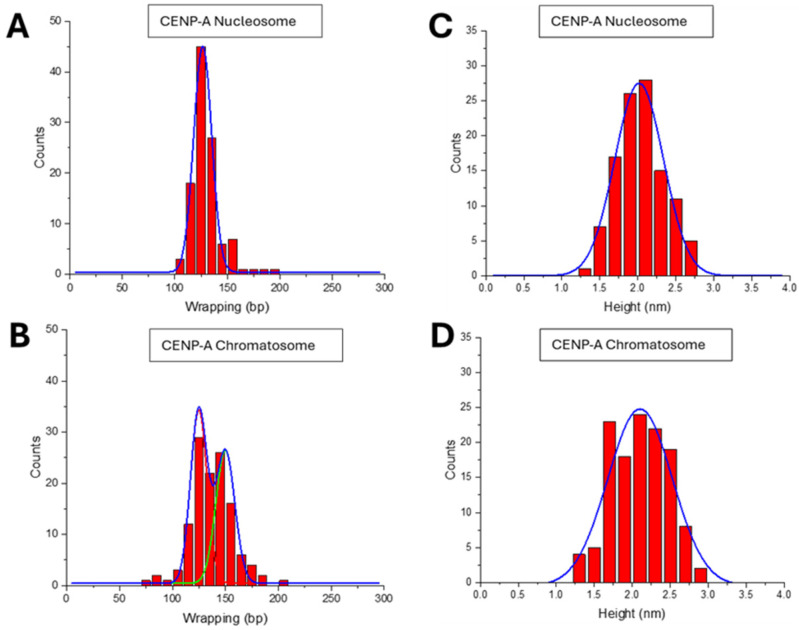
Results for the DNA wrapping lengths and the nucleosome particle sizes. (**A**) DNA wrapping length histograms for CENP-A nucleosomes (**A**, 127 ± 9 bp); (**B**) DNA wrapping length histograms for chromatosomes (150 ± 9 bp and 125 ± 8 bp). (**C**) Histograms of core particle height for CENP-A nucleosomes (2.0 ± 0.4 nm) and (**D**) histograms of core particle height for CENP-A chromatosomes (2.1 ± 0.5 nm), showing minimal changes upon H1 binding.

The DNA wrapping length for CENP-A nucleosomes was 127 ± 9 bp (Figure 7A). However, upon the incorporation of the H1 linker histone, two subpopulations emerged: ~47% of chromatosomes wrapped 150 ± 9 bp of DNA, while ~53% remained 125 ± 8 bp (Figure 7B). The difference in wrapping efficiency between CENP-A nucleosomes and chromatosomes was statistically significant, with a *p*-value of 0.007. These two subpopulations likely represent distinct structural states of CENP-A chromatosomes. The subpopulation with the lower wrapping length (125 ± 8 bp) corresponds to nucleosomes (i.e., no H1 histone in this population) that remain in a conformation similar to unmodified CENP-A nucleosomes, while the subpopulation with the increased wrapping length (150 ± 9 bp) reflects chromatosomes that have fully incorporated H1 histone. This dual population explains the gel electrophoresis data (Figure 5, lane 3), where a fraction of chromatosomes showed migration patterns consistent with nucleosome-like structures due to incomplete H1-mediated wrapping.

The height of the core particle was measured to assess the impact of H1 on nucleosome structure. CENP-A nucleosomes exhibited an average core particle height of 2.0 ± 0.4 nm (Figure 7C). CENP-A chromatosomes showed a slightly higher height value 2.1 ± 0.5 nm (Figure 7D), which correspond to *p* = 0.07, suggesting a difference in the core particle sizes for both species, although not highly significant.

**Figure 8:**
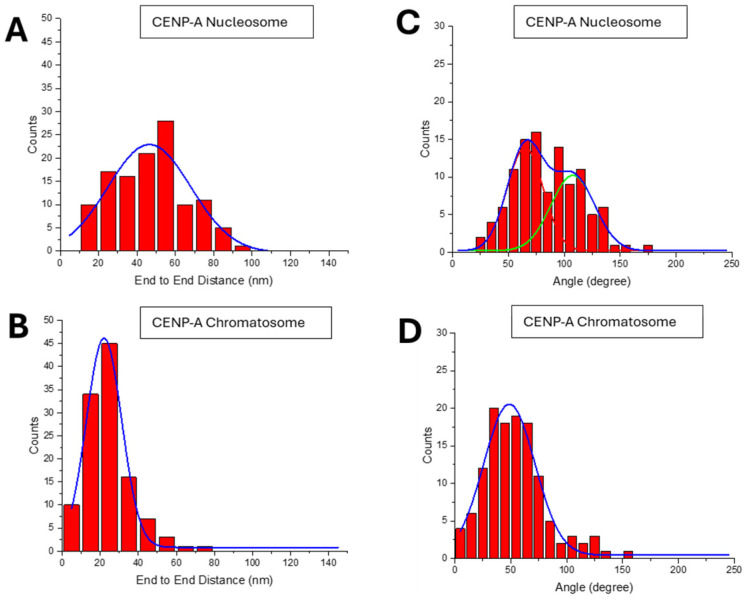
Results for the end-to-end distance and angle measurements: (**A**,**B**) End-to-end distance histograms for CENP-A nucleosomes (**A**, 46 ± 25 nm) and chromatosomes (**B**, 22 ± 11 nm). (**C**) DNA arm angle distributions for CENP-A nucleosomes; the bimodal distribution at 65 ± 16° and 108 ± 19° and (**D**) DNA arm angle distributions for CENP-A chromatosomes (49 ± 27°). H1 reduces DNA arm angles and promotes chromatosome compaction.

The end-to-end distance was measured to quantitatively characterize the compaction of CENP-A chromatosomes induced by H1 binding. For CENP-A nucleosomes, this distance in produced the mean value of 46 ± 25 nm (Figure 8A). In chromatosomes, the distribution moved to a much smaller value of 22 ± 11 nm (Figure 8B).

The angle between the arms was measured as well. The distribution for the CENP-A nucleosomes in Figure 8C was approximated with two Gaussians with values 65 ± 16° for 53% of the nucleosomes and 108 ± 19° for 47% of the nucleosome population. This variability in angles is visually illustrated by zoomed images (i)–(iii) of Figure 6A when the arms are quite far from the core. Results for the similar analysis for CENP-A chromatosomes are assembled in Figure 8D. They show that the distribution of the interarm angles is approximated with one Gaussian around the value 49 ± 27°. demonstrating that H1 compacts the DNA arms within chromatosomes.

## 3. Discussion

The results described above revealed a number of novel properties of complexes of nucleosomes with H1 linker histone, chromatosomes assembled spontaneously during the incubation of nucleosomes with H1.

According to the gel electrophoresis data for canonical nucleosomes (Figure 1B), the formation of chromatosomes is accompanied with the change of the mobility of both species. H3 chromatosomes form the major band with the mobility higher than the major population of H3 nucleosomes (band c in Figure 1B) suggesting that chromatosomes are more compact particles that the nucleosomes. Additionally, H1 histones stimulate assembly large assembles of chromatosomes not entering the gel (band “e” in Figure 1B). Finally, if the original sample of nucleosomes after the preparation for the gel, which includes the dilution of the sample spontaneously dissociates [52,53,54,55,56], chromatosome gel electrophoresis pattern did not reveal any free DNA, suggesting that compared with nucleosomes, chromatosomes are considerably stable. This finding is in line with publications [57,58,59].

**Figure 9:**
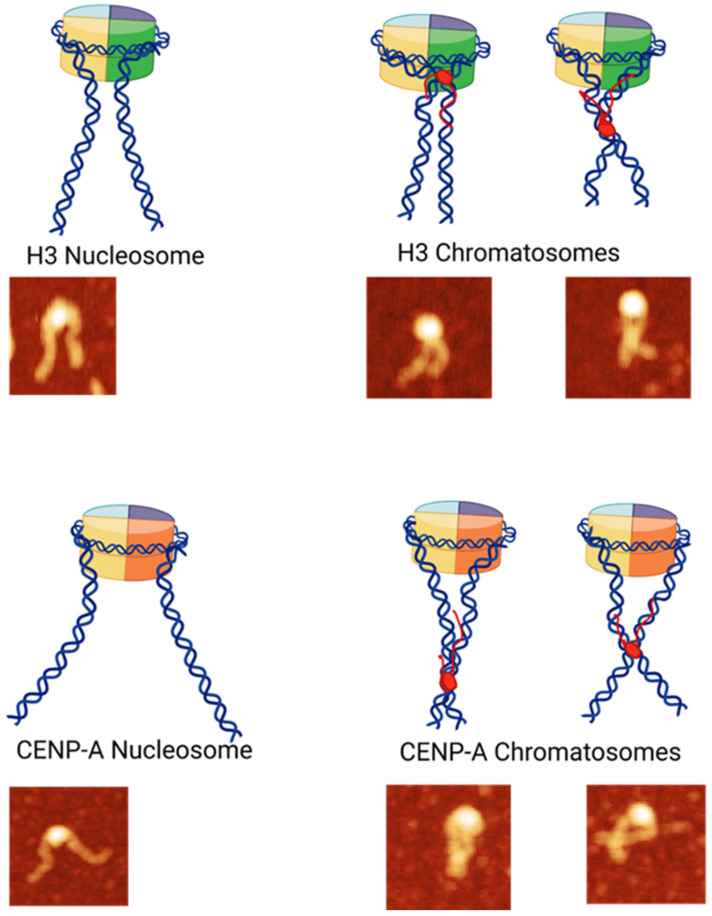
Schematic Representation of Nucleosomes and Chromatosomes (See Figure 2 and Figure 6). The cartoon was created using BioRender software (https://BioRender.com; accessed on 28 December 2024).

The effect of H1 histone on the shape of nucleosomes is seen visually in AFM images in Figure 2, which schematically are illustrated in Figure 9. This cartoon illustrates the conformational changes in nucleosomes upon the incorporation of the H1 linker histone, resulting in the formation of chromatosomes. Both non-cross and cross configurations of DNA arms are depicted to show distinct structural arrangements. These schematics provide a visual reference to aid in the interpretation of the analyzed AFM images and their corresponding morphologies.

Similar experiments with CENP-A nucleosomes did reveal the effect of H1 histone on the gel electrophoresis pattern but it is different from the data for H3 chromatosomes (Figure 1B and Figure 5). There are two dense bands b and c in which the position of band “c” coincides with that of CENP-A nucleosome suggesting that species “c” correspond to the CENP-A nucleosome sample without H1 bound. Indeed, CENP-A nucleosomes compared with canonical H3 ones, wraps ~120 bp DNA, so in CENP-A nucleosomes DNA makes 1.5 turns compared with ~1.7 turns for H3 nucleosomes [44,45,60,61,62] suggesting that this difference in the nucleosomes structure can contribute to the interaction of H1 histone with both types of nucleosomes, which can be clarified by the AFM nanoscale structural studies.

A few parameters characterizing the shape of nucleosomes were measured (Figure 3 and Figure 4) of the data for both types of nucleosomes and corresponding chromatosomes are assembled in Table 1.

First, the length DNA wrapped around the H3 histone core in H3 chromatosomes increases to 161 bp compared with 143 bp for nucleosomes. According to crystallographic [24] and Cryo-EM data [18],H1 histone binds to the nucleosome with ∼10 bp of DNA at both the entry and the exit sites of the nucleosome core particle. This structural feature is consistent with the ~20 bp increase of the length of DNA wrapped around the chromatosome found by AFM. Previous AFM studies have also shown that H1 binding increases DNA wrapping by a similar extent, supporting the conserved role of H1 in promoting chromatosome compaction [51].

Two other parameters, end-to-end distance and the angle between the DNA arms are consistent with this bridging effect of H1. Both are considerably lower compared with similar values for nucleosomes. However, AFM images revealed that H1 can bridge DNA arms far from the histone core. These are crossed chromatosomes and one of such examples is shown in frame (iii) in Figure 2B and Figure 9. The yield of such complexes is ~23.6% of the entire population of chromatosomes (Figure 2D). No such complexes were found in nucleosomes (Figure 2C). This finding suggests that H1 is capable of bridging of DNA strands, but control experiments did not reveal bridging of DNA molecules suggesting that close location of the DNA strands as it appears in nucleosomes is needed for the efficient bridging of DNA strands.

Stabilization of nucleosomes by H1 binding is another conclusion of the gel electrophoresis data (Figure 1B), and this conclusion is in line with the AFM imaging (Figure 2A,B and Figure 9). The ratio of free DNA in the chromatosome sample 10-fold less than in nucleosomes (Figure 2C,D). A spontaneous dissociation of a nucleosomes after their dilution to the nanomolar concentration range is a well-known phenomenon complicating single molecule studies [63,64]. The dissociation is rather rapid, in the seconds timescale, so the dissociation occurs during the dilution time, however, detergents such as 3-[(3-Cholamidopropyl)dimethylammonio]-l-propanesulfonate (CHAPS) stabilize the nucleosomes [48].

Formation of aggregates is another finding of gel electrophoresis data and multi choromatosome aggregates have been visualized with AFM (Figure S5). High-resolution images of two of such aggregates (i) and (ii) are shown to the right of the main scan. Clear internucleosomal contacts are seen in image (i), but interactions via DNA arms apparently bridging by the H1 histones can also be seen. Tri-nucleosome assembly in image (ii) is stabilized by the DNA bridging. The arrays of chromatosomes form liquid droplets [65], and we hypothesize that both molecular mechanisms contribute to the assembly of chromatosomes in aggregates. Although the internucleosomal interaction was observed in nucleosomal arrays [45,66], no nucleosomal aggregates were observed neither AFM nor in the gel electrophoresis experiments. The data in [67] suggests that histone tails contribute to the internucleosomal interactions, but the binding of H1 histone is the critical factor for the chromatosomes assembly in aggregates.

The interaction of H1 histone with CENP-A nucleosome qualitatively produces the same findings, although some features can be noted. Gel electrophoresis data (Figure 5 and Figure S4) revealed the high mobility band of CENP-A chromatosomes compared with the nucleosome ones, but there is another band with the mobility close to the one of CENP-A nucleosomes. The major structural feature of CENP-A nucleosomes is less size of the DNA wrapped around the core. According to the crystallographic [40] and Cryo-EM data [68], ~120 bp DNA is wrapped, which is close to 127 ± 9 bp value obtained in these experiments (Table 1). Structurally, this low value of wrapped DNA corresponds to 1.5 turns of DNA with the distance between the DNA arms as large as the nucleosome size. The DNA arms are much closer at the entry-exit segments of the DNA arms for canonical H3 nucleosomes, so in the crystal structure of chromatosome [24], H1 histone bridges the DNA arms in the ~10 bp proximity from the nucleosome core. There is no structural data for CENP-A chromatosomes, but it is reasonable to assume that the elevated distance between the DNA arms is a complicated factor for H1 binding to CENP-A nucleosomes. Indeed, the analysis of AFM images of CENP-A chromatosomes results in two peaks for the size of wrapped DNA (Figure 7B, Table 1). The left peak on the histogram corresponds to the DNA length 125 ± 8 bp, which coincides with 127 ± 9 bp value for CENP-A nucleosomes, suggesting that there a fraction of nucleosomes with structural parameters corresponding to those for CENP-A nucleosomes. This finding explains the correlation of the top band position with the one for CENP-A nucleosomes in Figure 5. The second peak in Figure 7B produces the value as large as 150 ± 9 bp, which similar to the H3 chromatosomes corresponds to the increase of the size of wrapped DNA due to binding of H1 histone to the nucleosome core. Nucleosomes are highly dynamic as reveled by single molecule studies including high-speed AFM [55,58,69], therefore, transiently CENP-A nucleosomes with elevated length of wrapped DNA can accommodate H1 histone to form chromatosomes in the structure similar to the one for H3 nucleosomes.

The formation of compact structures with characteristics similar to the ones for H3 chromatosomes is a direct effect of H1 assembly with CENP-A nucleosomes. There is a decrease in the end-to-end distances along with a small angle between the arms (Figure 8), which provide additional evidence for the CENP-A chromatosome assembly, their compaction compared with CENP-A nucleosomes. Formation of chromatosomes with crossed morphologies is another feature of CENP-A chromatosomes similar to the one for H3-chromatosomes.

Similar to H3 chromatosomes, CENP-A chromatosomes are much more stable compared with nucleosomes (Figure 6A). No free DNA is detected by the gel electrophoresis (Figure 5) and very few free DNA are identified with AFM (Figure 6C,D). Assembly in aggregates is also the property of CENP-A chromatosomes. One of such aggregates shown in frame (i) illustrates the extensive DNA bridging by H1 histones. Image (ii) illustrates the internucleosomal interaction leading to tight stacking of nucleosomes with the formation of a large particle (Figure S6). These novel features of chromatosome can contribute to their functional role.

H1 histone has long been recognized as a key player in the regulation of chromatin structure and function, with previous studies showing that H1 promotes chromatin compaction and gene silencing [70,71]. However, the detailed mechanisms by which H1 exerts these effects remain unclear. Our study provides direct evidence that H1 facilitates the wrapping of DNA around the histone core, stabilizes chromatin structure, and promotes the formation of higher-order chromatin structures through DNA-crossed arms (Figure 9). These results add to the growing body of evidence supporting the idea that H1 is not merely a structural component but also plays an active role in organizing chromatin at multiple levels [72,73].

The differential effects of H1 on canonical versus centromeric chromatin are of particular interest, as they suggest that H1 may have distinct roles depending on the chromatin context. In the case of centromeric chromatin, H1’s stabilizing effect may be crucial for maintaining the structural integrity of the centromere during cell division. This is especially relevant given that the centromere must remain stable to ensure proper chromosome segregation, and disruptions in centromeric chromatin structure can lead to aneuploidy and other chromosomal disorders [74,75,76,77]. Additionally, it is possible that H1’s effect on chromatin compaction could influence the accessibility of certain DNA regions, protecting centromeric sequences from unintended transcriptional events or damage during cell division, thereby contributing to the preservation of genomic stability [78,79,80]. Moreover, our findings on H1 bridging of separated nucleosomes support the idea that H1 may influence gene regulation by altering chromatin accessibility. The more compact chromatin observed in the presence of H1 likely reduces the accessibility of DNA to transcriptional machinery, thus promoting gene silencing. This idea is consistent with previous studies that have shown that the incorporation of H1 into chromatin correlates with reduced gene expression [19]. However, it is also possible that in specific regions of the genome, H1’s effects on chromatin might be more nuanced, potentially facilitating the formation of specialized chromatin states that are required for the expression of certain genes, such as those involved in stress response or cell cycle regulation [81,82,83,84].

Overall, both types of chromatosomes demonstrate similar structural features resulting to important properties with the elevated stability of nucleosomes as one of them. This finding suggests that such functions of chromatin as transcription and replication is regulated by H1 histone dissociation of which should simply these processes. Additionally, elevated interaction between chromatosomes, should contribute to the chromatin assembly, so H1 plays a critical role in the dynamics of chromatin, which is another factor for the regulation of physiological properties of chromatin.

## 4. Materials and Methods

### 4.1. Preparation of DNA Substrate

The DNA construct was prepared the same way we had previously prepared [49]. The DNA substrates for nucleosome assembly were produced via PCR using a pUC57 plasmid vector from BioBasic (Markham, ON, Canada) as the template. A DNA construct was prepared for the substrates: containing 147 bp of the strong positioning Widom 601 sequence flanked by 113 bp and 117 bp of plasmid DNA (Figure 1A). Following PCR amplification, the DNA substrate was concentrated and purified using the Qiagen Gel Extraction Kit (Hilden, DE, USA). The DNA concentration was then determined using a NanoDrop Spectrophotometer (ND-1000, Thermo Fisher, Waltham, MA, USA).

### 4.2. Nucleosome & Chromatosome Assembly

#### 4.2.1. Canonical H3 Nucleosomes and Chromatosomes

The nucleosome assembly involves a gradual dialysis process, transitioning from a high salt concentration (2 M) to a lower concentration (250 mM) [85]. H3 histone octamers, obtained from The Histone Source (Fort Collins, CO, USA), were mixed with purified DNA substrates at a 1:1 DNA/histone octamer molar ratio. The mixture was placed in a Slide-A-Lyzer MINI dialysis unit (2000 MWCO, Thermo Fisher Scientific) and dialyzed at 4 °C, with the 2 M NaCl buffer being gradually replaced by a 250 mM NaCl buffer. A peristaltic pump facilitated this exchange, introducing the secondary buffer (250 mM NaCl, 1 mM EDTA (pH 8), 10 mM Tris (pH 7.5), 2 mM mercaptoethanol) while simultaneously removing the initial buffer (2 M NaCl, 1 mM EDTA (pH 8), 10 mM Tris (pH 7.5), 2 mM mercaptoethanol), keeping the volume constant. The NaCl concentration was adjusted gradually over 20 h at 4 °C, followed by an additional hour of incubation in the secondary buffer to ensure a final concentration of 250 mM NaCl. For chromatosome reconstruction, The human H1.0 linker histone, obtained from The Histone Source (Fort Collins, CO, USA), was incorporated with nucleosomes at a 1:2 molar ratio (nucleosome to H1 histone) through a dialysis step as described in [86]. The process was carried out in HE buffer (10 mM HEPES, pH 7.4, and 0.1 mM EDTA) for 2 h at 4 °C.

#### 4.2.2. Centromeric CENP-A Nucleosomes and Chromatosomes

The assembly of CENP-A nucleosomes followed the protocol for H3 nucleosome assembly, including an extra step in which the CENP-A/H4 tetramer and H2A/H2B dimer are combined in a 1:2 molar ratio to form the correct octamer assembly. These components are sourced from EpiCypher (Durham, NC, USA). The protocol for the assembly of chromatosomes was the same as that described above for H3 chromatosomes.

### 4.3. Atomic Force Microscopy Imaging in Air

A 167 μM solution of 1-(3-aminopropyl)-silatrane (APS) was used to modify freshly cleaved mica for 30 min as previously described [64,86,87]. The nucleosome stock solution was diluted to a final concentration of 2 nM (based on DNA concentration) in 10 mM HEPES buffer, followed by deposition onto APS mica. The sample on the mica was allowed to incubate for 2 min, then gently rinsed with deionized water and dried under a slow argon flow. Subsequently, the samples were placed under vacuum to dry overnight. Imaging was carried out using a MultiMode AFM/NanoScope V with the use of TESPA probes (Bruker Nano Inc., Camarilla, CA, USA), capturing images over area of 1000 nm × 1000 nm with 1024 pixels per line.

### 4.4. 5% Polyacrylamide Native-PAGE

Both types of nucleosomes and chromatosomes were analyzed using 5% polyacrylamide native gel electrophoresis (Native-PAGE) [88,89]. A 30% acrylamide (29:1) solution was prepared, and the gel was run in 1× TBE buffer (90 mM Tris base, 90 mM boric acid, 2 mM EDTA). Samples were loaded onto the gel, and electrophoresis was conducted at 35 V (5 V/cm) until sufficient separation was achieved. The gel was stained with ethidium bromide (EtBr) at a final concentration of 0.5 µg/mL to visualize the complexes. The gels were scan through ImageQuant LAS 4000 (GE Healthcare).

### 4.5. Data Analysis

Data analysis was carried out using established methods previously validated in our lab [90]. Contour length measurements were performed with Femtoscan (Advance Technologies Center, Moscow, Russia), starting from the end of the DNA strand and extending to the center of the nucleosome, followed by measuring the opposite DNA arm. To account for the histone core, 5 nm was subtracted from each DNA arm measurement (Figure S7). The lengths, initially recorded in nanometers, were converted to base pairs (bp) using a conversion factor derived by analyzing the contour length of naked DNA in each image. This conversion factor, typically around 0.34 nm/bp, was obtained by dividing the total length of naked DNA by its known base pair count. Height measurements were conducted in Femtoscan through grain analysis, with each nucleosome selected individually and assessed using multiple cross-sections. Additionally, the angles formed by the entry and exit sites of each nucleosomal core particles were measured, and the end-to-end distance was determined by measuring the distance between the ends of the two DNA arms (Figure S7). Following DNA measurements, Origin software (Version 2016, OriginLab Corporation, Northampton, MA, USA) was used to bin the data, create histograms, and determine mean Gaussian distributions. The error associated with mean is standard deviation. Gel images were analyzed by GelAnalyzer 23.1.1 software developed by Istvan Lazar Jr., PhD and Istvan Lazar Sr., PhD, CSc (available at www.gelanalyzer.com; accessed on 1 December 2024). 

## Figures and Tables

**Table 1:** Structural features of H3 and CENP-A nucleosomes and chromatosomes.

	H3		CENP-A	
Parameter	Nucleosome	Chromatosome	Nucleosome	Chromatosome
Wrapping (bp)	143 ± 16 bp	161 ± 21 bp	127 ± 9 bp	125 ± 8 bp
				150 ± 9 bp
End-to-End distance (nm)	52 ± 22 nm	24 ±13 nm	46 ± 25 nm	21 ± 11 nm
Angle between the arms (degrees)	90 ± 35°	38 ± 18°	65 ± 16°	49 ± 27 °
			108 ± 19°	

## Data Availability

All data are included in this paper.

## Supplementary Materials

The following supporting information can be downloaded at: www.mdpi.com/article/10.3390/ijms26010303/s1.
