# Supplementary material for: Nanoscale Characterization of Interaction of Nucleosomes with H1 Linker Histone
Source: Int J Mol Sci. 2024 Dec 31;26(1):303. doi: 10.3390/ijms26010303 (PMC11719560; doi:10.3390/ijms26010303)
Supplement: Supplementary file 1 [file ijms-26-00303-s001.zip › ijms-3390512-supplementary.pdf]

Nanoscale Characterization of Interaction of Nucleosomes with H1 Linker Histone

Ahmed Yesvi Rafa, Shaun Filliaux and Yuri L. Lyubchenko*

Supplementary figures

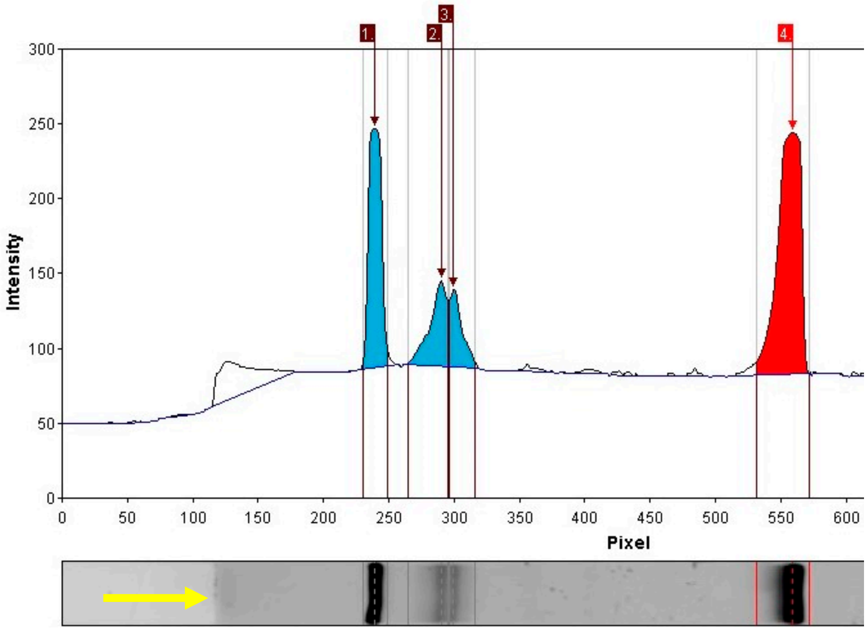

Supplemental Figure S1: Scan (top) of the gel of H3 nucleosome (bottom, raw image): The red-shaded area represents free DNA, while the blue-shaded area corresponds to nucleosomes.

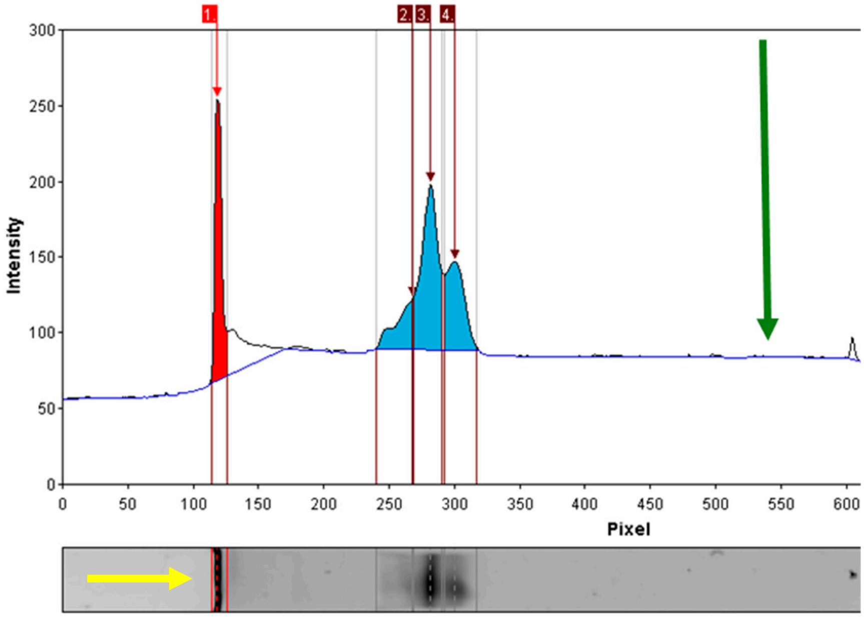

Supplemental Figure S2: Scan (top) of the gel of H3 chromatosome sample (bottom, raw image) The blue-shaded area represents chromatosomes, and the red-shaded area indicates large aggregates formed by chromatosomes. The green arrow marks the position of the free DNA band observed in the H3 nucleosome gel (Figure S1). The yellow arrows indicate the starting point of the gel.

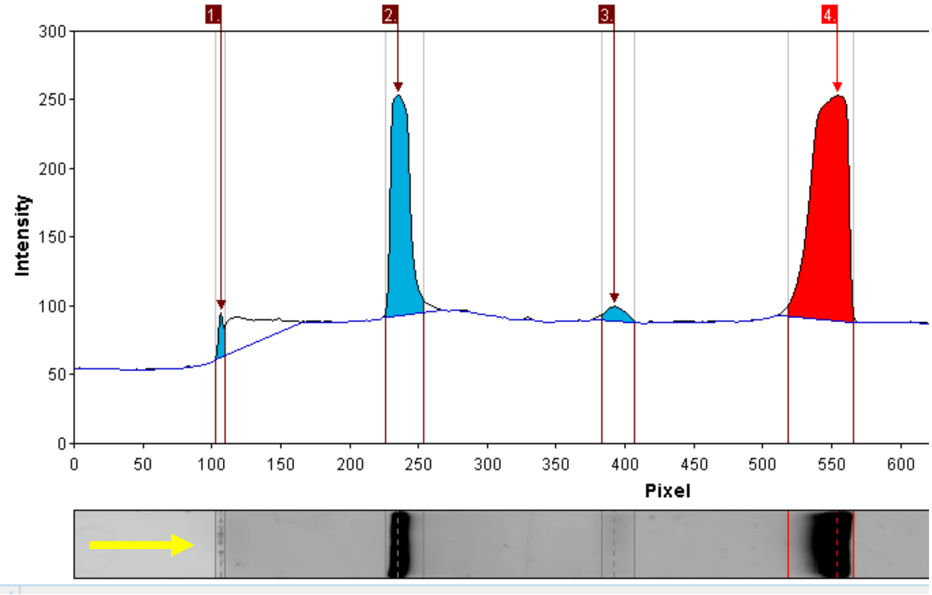

Supplemental Figure S3: Scan (top) of the gel of CENP-A nucleosome sample (bottom raw image) The blue-shaded area corresponds to nucleosome, and the red-shaded area represents free DNA.

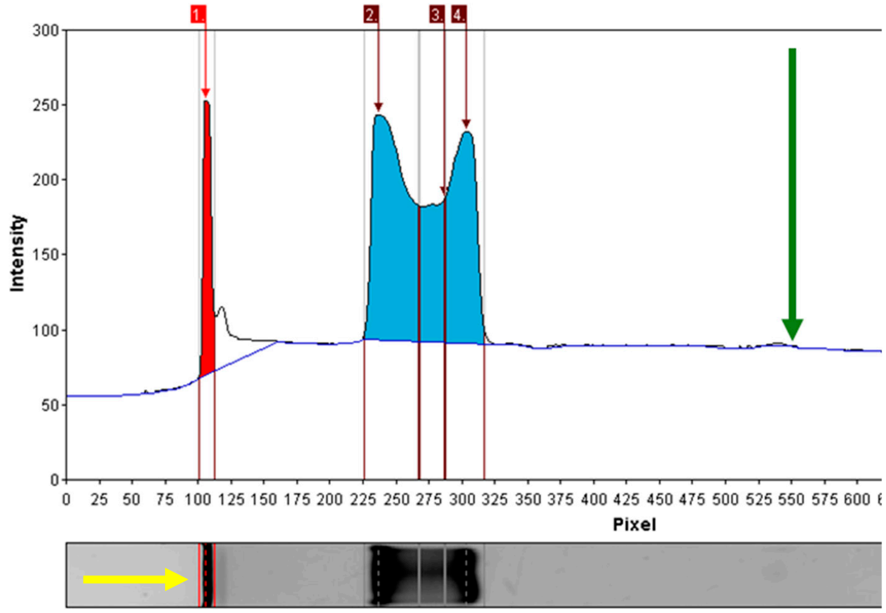

Supplemental Figure S4: Scan (top) of the gel of CENP-A chromatosome sample (bottom, raw image) The blue-shaded area corresponds to chromatosomes, while the red-shaded area indicates large aggregates. The green arrow marks the position of the free DNA band observed in the H3 nucleosome gel (Figure S3). The yellow arrows indicate the starting point of the gel.

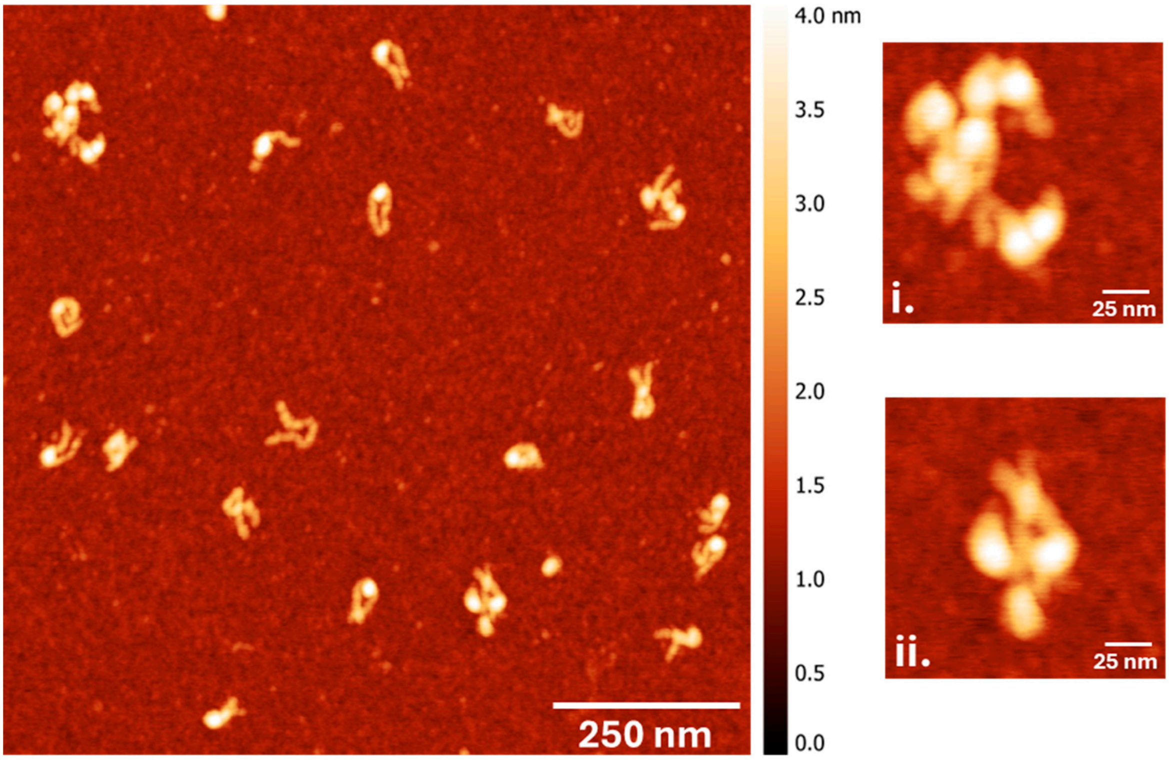

Supplemental Figure S5AFM images of aggregates formed by H3 chromatosomes: The image highlights DNA bridging and internucleosomal interactions mediated by H1 histone. Zoomed image (i) are larger aggregate and zoomed image (ii) are smaller aggregate.

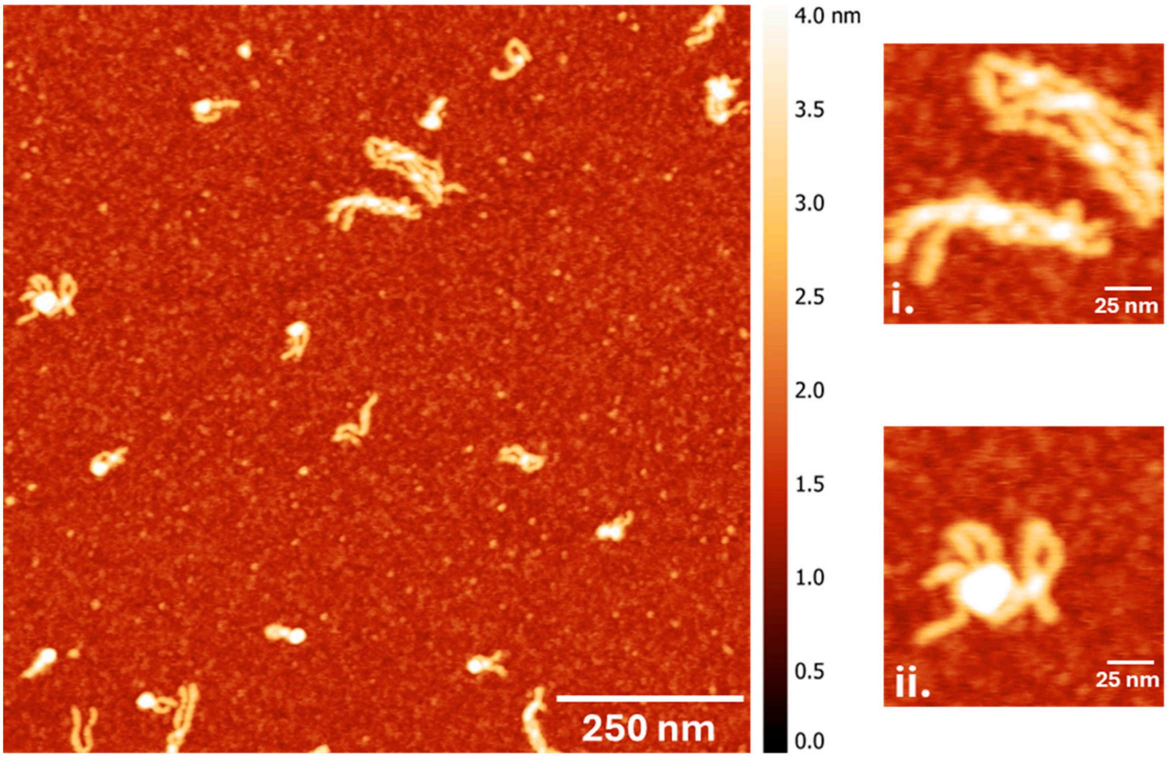

Supplemental Figure S6 AFM images of aggregates formed by CENP-A chromatosomes: The image shows compact assemblies stabilized by DNA bridging and histone-mediated interactions.

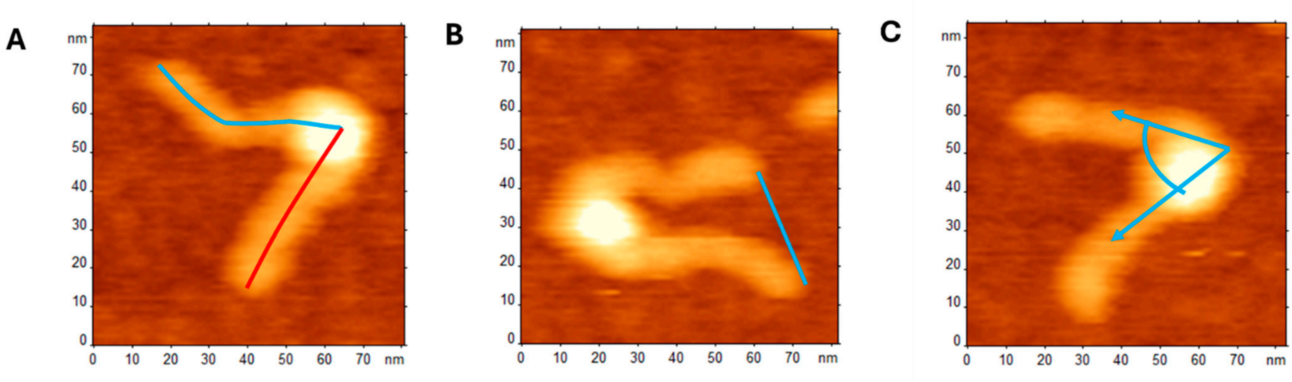

Supplemental Figure S7Analysis of AFM Images: (A) The image showed the measurement of DNA linker arms for determining the wrapping efficiency. The blue line (L_1) is 46 nm, and the red line (L_2) is 43 nm. To account for the histone core, 5 nm was subtracted from the measurement of each DNA arm. The total length of DNA is 132 nm ($377\ bp\ *\ 0.35\frac{nm}{bp}$). The conversion coefficient determined from AFM measurements of the DNA contour length is 0.35 nm/bp. The wrapping efficiency (the length of wrapped DNA) is calculated as follows:

$$Wrapping\ Efficiency\ = Total\ DNA\ Length\ (nm) - (L_1 + L_2 - 10)\ nm \Rightarrow$$
$$Wrapping\ Efficiency\ (nm) = 132 - (46 + 43 - 10) = 53nm$$

$$Wrapping\ Efficiency\ (bp) = \frac{Wrapping\ Efficiency\ (nm)}{0.35\ (\frac{nm}{bp})} = \frac{53\ (nm)}{0.35\ (\frac{nm}{bp})} = 151\ bp$$

(B) This AFM image illustrates the measurement of the distance between the two DNA arms, yielding to the end-to-end distance of 30 nm.

(C) This depicts the angle measurement between the two DNA arms, which is determined to be 57°.
